# Identification of autophagy receptors for the Crohn’s disease-associated adherent-invasive *Escherichia coli*

**DOI:** 10.3389/fcimb.2024.1268243

**Published:** 2024-03-28

**Authors:** Alison Da Silva, Guillaume Dalmasso, Anaïs Larabi, My Hanh Thi Hoang, Elisabeth Billard, Nicolas Barnich, Hang Thi Thu Nguyen

**Affiliations:** ^1^ M2iSH (Microbes, Intestine, Inflammation and Susceptibility of the Host), UMR 1071 Inserm, Université Clermont Auvergne, INRAE USC 1382, CNRH, Clermont-Ferrand, France; ^2^ Department of Cell Biology, Faculty of Biology, University of Science, Vietnam National University, Hanoi, Vietnam

**Keywords:** adherent-invasive *E. coli* (AIEC), autophagy, Crohn’s disease, p62, NDP52

## Abstract

**Introduction:**

Crohn’s disease (CD) is a chronic inflammatory bowel disease, of which the etiology involves genetic, environmental and microbial factors. Adherent-invasive *Escherichia coli* (AIEC) and polymorphisms in autophagy-related genes have been implicated in CD etiology. Autophagy is a key process for the maintenance of cellular homeostasis, which allows the degradation of damaged cytoplasmic components and pathogens via lysosome. We have shown that a functional autophagy is necessary for AIEC clearance. Here, we aimed at identifying the autophagy receptor(s) responsible to target AIEC to autophagy for degradation.

**Methods:**

The levels of autophagy receptors p62, NDP52, NBR1, TAX1BP1 and Optineurin were knocked down in human intestinal epithelial cells T84 using siRNAs. The NDP52 knock-out (KO) and p62 KO HeLa cells, as well as NDP52 KO HeLa cells expressing the wild-type NDP52 or the mutated *NDP52^Val248Ala^* protein were used.

**Results and discussion:**

We showed that, among the tested autophagy receptors (p62, NDP52, NBR1, TAX1BP1 and Optineurin), diminished expression of p62 or NDP52 increased the number of the clinical AIEC LF82 strain inside epithelial cells. This was associated with increased pro-inflammatory cytokine production. Moreover, p62 or NDP52 directly colocalized with AIEC LF82 and LC3, an autophagy marker. As the *NDP52^Val248Ala^* polymorphism has been associated with increased CD susceptibility, we investigated its impact on AIEC control. However, in HeLa cell and under our experimental condition, no effect of this polymorphism neither on AIEC LF82 intracellular number nor on pro-inflammatory cytokine production was observed. Together, our results suggest that p62 and NDP52 act as autophagy receptors for AIEC recognition, controlling AIEC intracellular replication and inflammation.

## Introduction

Crohn’s disease (CD) is an inflammatory bowel disease (IBD) characterized by chronic inflammation of any part of the gastrointestinal tract (Torres et al., 2017). Evidence has shown that CD resulted from a complex interplay between environmental factors and genetic susceptibility, leading to dysregulated immune responses against the intestinal microbiota (Carrière et al., 2014). To date, no curative treatment is available for CD patients, and only symptomatic treatments are proposed to limit the intensity and the frequency of the inflammatory flare (Torres et al., 2017). This indicates a need to fully elucidate the underlying causes of the disease.

Intestinal dysbiosis with altered composition and diversity of the microbiota has been consistently described in patients with IBD (Carrière et al., 2014). It has been shown that the intestinal mucosa of CD patients is abnormally colonized by adherent-invasive *Escherichia coli* (AIEC) pathobionts (Darfeuille-Michaud et al., 1998, 2004). AIEC bacteria are characterized by their abilities to adhere to and to invade intestinal epithelial cells, to survive and replicate within macrophages without inducing cell death, and to induce the release of pro-inflammatory cytokines (Larabi et al., 2020a). Furthermore, our group has shown that the replication ability of AIEC is increased in macrophages from CD patients compared to healthy individuals (Vazeille et al., 2015), and that the defect in AIEC clearance of CD patients-derived macrophages is linked to the polymorphisms in autophagy-related genes, including *IRGM* (Immunity-related GTPase family M protein) and *ULK-1* (Unc-51 Like Autophagy Activating Kinase 1) (Buisson et al., 2019). Our recent study showed that AIEC are involved in the early stages of ileal lesions in CD, and the presence of AIEC within surgical specimen is predictive of endoscopic post-operative recurrence at 6 months (Buisson et al., 2023). This study strongly demonstrates the clinical relevance of AIEC implication in the etiopathogenesis of CD.

Macroautophagy (hereafter referred to as autophagy) is a tightly regulated and conserved catabolic process by which the cell degrades cytoplasmic components, such as misfolded proteins, damaged organelles or invasive pathogens, via lysosome (Klionsky et al., 2021). Briefly, autophagy is initiated by the formation of the isolated double-membrane phagophore that elongates and matures to form an autophagosome. Autophagosome then fuses with lysosome for the subsequent degradation of its content (Klionsky et al., 2021). Our group has shown that upon AIEC infection, autophagy is induced in host cells, and a functional autophagy is necessary to limit the intracellular replication of AIEC (Lapaquette et al., 2010, 2012; Bretin et al., 2016, 2018; Dalmasso et al., 2021). We also showed that while host cells induce a functional autophagy to eliminate AIEC, AIEC can subvert autophagy by up-regulating the levels of microRNAs 130a and 30c of host cells (Nguyen et al., 2014) or by impairing host SUMOylation, a eukaryotic reversible post-translational modification, in which SUMO, a ubiquitin-like polypeptide, is covalently linked to target proteins (Dalmasso et al., 2019). This consequently leads to abnormal AIEC intracellular replication and enhanced AIEC-induced inflammation (Nguyen et al., 2014; Dalmasso et al., 2019). Furthermore, upon AIEC infection, host cells secrete exosomes (Carrière et al., 2016), extracellular vesicles of 30-100 nm, that can transfer miR-30c and miR-130a from cell-to-cell to inhibit autophagy, favoring AIEC intracellular replication (Larabi et al., 2020b). Importantly, we and others have shown that the CD-associated SNPs in autophagy-related genes, including *ATG16L1*, *IRGM* and *NOD2*, lead to impaired autophagy, resulting in abnormal AIEC intracellular replication and increased secretion of pro-inflammatory cytokines (Nguyen et al., 2013; Larabi et al., 2020a).

Autophagy can be non-selective, where bulky portions of the cytoplasm are degraded upon stress, or highly selective, by which pre-selected and specific cellular components are degraded. In selective autophagy, the first step involves the ubiquitination of the cargo, following which it gets recognized by autophagy receptors for subsequent targeting into an autophagosome, which matures into a degradative vesicle after fusion with lysosome (Lamark and Johansen, 2021). Selective autophagy is classified according to the targeted cargo: mitophagy for mitochondria, pexophagy for peroxisomes, ribophagy for ribosomes, lipophagy for lipid droplets, aggrephagy for aggregated proteins and xenophagy for invading pathogens (Lamark and Johansen, 2021). Ubiquitinated intracellular bacteria could be recognized by multiple ubiquitin-binding cargo receptors, such as Optineurin (Wild et al., 2011), NBR1 (Kirkin et al., 2009; Waters et al., 2009), TAX1BP1 (Tumbarello et al., 2015), p62 (Bjørkøy et al., 2005; Pankiv et al., 2007) and NDP52 (Thurston et al., 2009).

p62 (also known as SQSTM1 (sequestosome 1)) is the first selective autophagy receptor to be identified and described (Bjørkøy et al., 2005; Pankiv et al., 2007). It is involved in cellular stress response, clearance of protein aggregates, defective organelles as well as invading pathogens (Sharma et al., 2018; Vainshtein and Grumati, 2020). Indeed, the importance of p62 in xenophagy was primarily explored in the control of the invading bacteria *Salmonella enterica* serovar Typhimurium (Zheng et al., 2009). Other bacterial species, such as *Shigella flexneri* (Mostowy et al., 2011), *Listeria monocytogenes* (Mostowy et al., 2011) and *Mycobacterium tuberculosis* (Franco et al., 2017) have been also reported to be selectively targeted by p62 for recruitment and delivery into nascent LC3-positive autophagosomes. p62 acts with other receptors such as NDP52 (nuclear dot protein 52 kDa, also known as CALCOCO2) to target *S.* Typhimurium (Cemma et al., 2011), *Listeria monocytogenes* (Mostowy et al., 2011) and *Shigella flexneri* (Mostowy et al., 2011) to autophagosomes.

NDP52 is an important selective autophagy receptor which is involved in the maintenance of cellular homeostasis by degrading damaged mitochondria via mitophagy (Heo et al., 2015). It also plays an essential role in xenophagy. Indeed, NDP52 targets various infectious pathogens such as *Streptococcus pyogenes* (Von Muhlinen et al., 2010), *S.* Typhimurium (Von Muhlinen et al., 2010) and *Shigella flexneri* (Mostowy et al., 2011) for their selective degradation by xenophagy. NDP52 uses its C-terminal ubiquitin-binding zinc finger domain (UBZ) to recognize ubiquitin on the bacterial surface and the LC3-interacting region (LIR) for the binding with LC3 molecules on autophagosome, facilitating the recruitment of autophagic machinery surrounding the pathogens (Ivanov and Roy, 2009; Thurston et al., 2009). Moreover, NDP52 also takes part in reducing inflammation via down-regulating the NF-κB (nuclear factor-kappa B) signaling (Inomata et al., 2012; Ellinghaus et al., 2013). In 2013, a whole exome sequencing study identified an association between CD susceptibility and a common missense variant, Val248Ala (SNP rs2303015), which leads to the replacement of valine by alanine at position 248 of the NDP52 protein (Ellinghaus et al., 2013). Ellinghaus and colleagues reported that while the wild-type *NDP52* was able to decrease NF-κB activation in response to poly(I:C), a TLR (Toll-like receptor) agonist, in HeLa cells, the CD-associated *NDP52^Val248Ala^* variant failed to do so (Ellinghaus et al., 2013). Thus, the negative feedback on TLR signaling exerted by NDP52 was impaired by the *NDP52^Val248Ala^* variant, and this could be one of the mechanisms underlying the implication of this variant in CD pathogenesis (Ellinghaus et al., 2013). Nevertheless, the impact of this risk variant on autophagy targeting bacteria or xenophagy has not been investigated. So far, the autophagy receptors responsible for the recognition of AIEC and targeting the bacteria to autophagosome have not yet been identified. In this study, we aimed to identify the autophagy receptor(s) specific for AIEC targeting and to investigate the impact of the CD-associated *NDP52^Val248Ala^* variant on host response to AIEC infection.

## Materials and methods

### Bacterial strains

The AIEC reference strain LF82 was isolated from a chronic ileal lesion of a CD patient (Darfeuille-Michaud et al., 1998). The clinical AIEC strains CEA501S and CEA614S were isolated from the ileal lesions of CD patients from the CEALIVE cohort (Buisson et al., 2021). The LF82-GFP bacteria was used to visualize the bacteria by fluorescent microscopy as previously described (Nguyen et al., 2014; Bretin et al., 2016). The mCherry-labeled AIEC LF82 strain was obtained by bacterial conjugation in solid medium. Briefly, the mCherry donor strain, the AIEC LF82 recipient strain and the strain carrying the transposase were grown overnight in Luria-Broth (LB) with corresponding antibiotics at 37°C. These 3 strains were mixed together, centrifuged for 2 min at 1,900 *g*, re-suspended in 1.5 mL of LB to eliminate antibiotics and centrifuged again. Then, the pellet was re-suspended in 50 µL of LB, and this solution was filtered through a 0.45 µm filter membrane on LB agar plate. The plate was incubated for 2 to 6 hours at 37°C, and then, the filter membrane was transferred in a 50 mL tube to resuspend the bacteria in 1 mL of LB. It was spread on LB agar plates and incubated overnight at 37°C. The *E. coli* K12 C600 strain (Appleyard, 1954) was used as a non-invasive strain. All bacterial strains were grown overnight in LB at 37°C without agitation.

### Cell lines and culture conditions

The NDP52 knock-out (KO) and p62 KO HeLa cells, as well as their corresponding control HeLa cells, which express NDP52 or p62, respectively, were kindly provided by Prof. Richard J. Youle (National Institutes of Health, Bethesda, Maryland, USA) (Lazarou et al., 2015). HeLa cells expressing the GFP-LC3 construct was kindly given by Drs. Aurore Rozières and Christophe Viret (CIRI, Centre International de Recherche en Infectiologie, Lyon, France). HeLa cells were cultured in Dulbecco’s Modified Eagle Medium (DMEM) with L-glutamine (Gibco) supplemented with 10% fetal bovine serum (FBS) (Dutscher) and 1% antibiotic/antimycotic solution (Penicillin G/Streptomycin/Amphotericin B) (Cytiva). The intestinal epithelial cell line T84 (ATCC, CCL-248) was cultured in DMEM/F-12 Nutrient Mixture (Ham) (Gibco) supplemented with 10% FBS, 1% Glutamine (Gibco), 1% antibiotic/antimycotic solution (Penicillin G/Streptomycin/Amphotericin B) and 1% Hepes (Dutscher). All cells were maintained in an atmosphere with saturated humidity and containing 5% CO_2_ at 37°C.

### Plasmid construction

The constructs expressing wild-type NDP52, or mutated NDP52^Val248Ala^, or NDP52-GFP or NDP52^Val248Ala^-GFP were generated. NDP52 cDNA was cloned from T84 cells into the pEGFP-C2 vector (Clontech) using the SuperFi II DNA polymerase (Invitrogen) and the following primers:

NDP52_Forward-EcoRI_: 5’-GGGCGAATTCTATGGAGGAGACCATCAAAG-3’;
NDP52_Reverse-BamHI_: 5’-GTTGGATCCTCAGAGAGAGTGGCAGAACACG-3’.
The valine at position 248 was replaced by an alanine by changing a C for a T at the position 743 of NDP52 cDNA using the Site-Directed Mutagenesis Kit QuickChange II (Agilent) and the following primers:
Forward: 5’-GAGAAAGAAATGGAGAAGCTTGCTCAGGGAGATCAAGATAAGAC-3’;
Reverse: 5’-GTCTTATCTTGATCTCCCTGAGCAAGCTTCTCCATTTCTTTCTC-3’.
GFP was removed from the pEGFP-C2-NDP52 construct using the In-Fusion HD Cloning Kit (Takara) and the following primers:
Forward: 5’-TCGCCACCATAACTGATCATAATCAGCCATACC-3’;
Reverse: 5’-CAGTTATGGTGGCGACCGGTAGCGC-3’.
All the sequences were verified by DNA sequencing (Eurofins Genomics).

### Transfection

All transfections were performed using Lipofectamine 3000 RNAimax (Invitrogen) according to the manufacturer’s instructions. Briefly, T84 cells or NDP52 KO HeLa cells were seeded in 24-well tissue culture plates. The following day, lipofectamine 3000 reagent was diluted in Opti-MEM medium (Gibco), and nucleotide mix was prepared by mixing siRNAs (Dharmacon ON-TARGETplus SMARTPool; final concentration: 50 nM) against NDP52 (#L-010637-00-0005), Optineurin (#L-016269-00-0005), p62 (#L-010230-00-0005), NBR1 (#L-010522-00-0005) or TAX1BP1 (#L-016892-00-0005) or 500 ng of plasmid in Opti-MEM medium. Then, the lipofectamine 3000 reagent was added to the nucleotide mix and incubated for 5 min at room temperature. The mixed solution was added to the cells and incubated at 37°C in an atmosphere containing 5% CO_2_. After 6 h, the transfection medium was removed, cells were washed once with PBS and incubated with antibiotic-free culture medium.

### Invasion assay

Bacterial strains were cultured overnight in LB at 37°C without agitation. Bacterial concentrations were estimated by measuring the optical density at 600 nm. Bacteria were re-suspended in a proper volume of fresh antibiotic-free culture medium to allow infection of host cells at a MOI (multiplicity of infection) of 10, 100 or 200. The number of intracellular bacteria was determined using the gentamicin protection assay as we described previously (Nguyen et al., 2014; Bretin et al., 2016). Briefly, cell monolayers were incubated with bacteria for 3 h, washed for three times with PBS 1X and then incubated with antibiotic-free culture medium containing 100 µg/mL of gentamicin for the indicated time. To determine the number of intracellular bacteria, cell monolayers were washed once with PBS and lysed with 1% Triton X-100 (Euromedex) in deionized water. Samples were serially diluted and plated onto LB agar plates to determine the number of CFU (colony-forming unit) of the bacteria.

### Immunofluorescence analysis

GFP-LC3-expressing HeLa cells were seeded on coverslips in 24-well tissue culture plates 2 days before infection. Cells were infected with AIEC LF82-mCherry at a MOI of 100 and centrifugated at 160 g for 10 min to promote the contact between the bacteria and the cells. The cells were then incubated with the bacteria for 3 h at 37°C in an atmosphere containing 5% CO_2_. Cells were then washed three times with PBS 1X and incubated with antibiotic-free culture media containing 100 µg/mL of gentamicin for the indicated time. Then, cells were fixed with 3.7% paraformaldhehyde (PFA) (Sigma) in PBS 1X at room temperature for 10 min, and then permeabilized with 0.1% Triton X-100 in PBS 1X at room temperature for 10 min. Cells were incubated with the saturation buffer (PBS 1X containing 5% FBS, 3% Bovine Serum Albumin (BSA) and 0.025% Triton X-100) at room temperature for 1 h. Cells were then incubated overnight at 4°C with a primary antibody diluted in the saturation buffer (rabbit anti-NDP52 antibody, #ab68588, Abcam, dilution: 1/100) in a humid box. The following day, after 6 washes with PBS 1X, cells were incubated with the corresponding secondary antibody diluted in the saturation buffer (anti-rabbit Cy5, #A10523, Invitrogen, dilution: 1/400) in a humid box for 45 min. Nuclei were stained with Hoescht. Coverslips were mounted with a Mowiol solution (Interchim). Images were taken using Zeiss LSM 800 with Airyscan confocal microscope.

### Flow cytometry

NDP52 KO HeLa cells were seeded in 6-well tissue culture plates. The following day, cells were transfected with wild-type NDP52-GFP or NDP52^Val248Ala^-GFP constructs, or the empty plasmid. Then, cells were infected with AIEC LF82-mCherry at a MOI of 100 for 3 h. Cells were then washed three times with PBS 1X and incubated with antibiotic-free culture media containing 100 µg/mL of gentamicin for 1 h. Cells were trypsinized, resuspended in fresh antibiotic-free culture media and centrifugated for 5 min at 130 g. The cells were finally resuspended in PBS 1X containing 1% BSA and 2 mM EDTA. The flow cytometry analysis was performed on Attune flow cytometer (Life Technologies) using the Attune NXT software.

### Enzyme-linked immunosorbent assay

Cell culture supernatants were collected at different time points during invasion assay (before infection and at 4 h, 10 h and 24 h post-infection). The amounts of secreted IL-8 and IL-6 levels were determined by enzyme-linked immunosorbent assay (ELISA) according to the manufacturer’s instructions (Duoset, R&D systems).

### Immunoblotting analysis

Cells were washed with PBS 1X and lysed with RIPA buffer (20 mM Tris-HCl, 150 mM NaCl, 2 mM EDTA, 1% NP-40, pH 7.4) containing protease and phosphatase inhibitor cocktail (Roche) and 20 mM Na-orthovanadate. Lysates were recovered in an eppendorf tube and disrupted for 6 min at 4°C. Then, lysates were centrifuged at 14,000 *g* for 15 min at 4°C, and the supernatants were used for immunoblotting. Protein concentrations were measured using the DC protein assay kit (Bio-Rad). The samples were diluted with 4X Laemmli sample buffer (277.8 mM Tris-HCl, pH 6.8, 44.4% (v/v) glycerol, 4.4% SDS, 0.02% bromophenol blue; Bio-Rad) containing β-mercaptoethanol, followed by heating for 5 min at 95°C. Whole-cell lysates were separated by acrylamide gel electrophoresis (10 µg of proteins per well), and transferred to a nitrocellulose membrane (Amersham™ Protran™ supported 0.45 µm). The membrane was then incubated with the blocking buffer (PBS 1X containing 0.1% Tween 20 and 5% BSA) for 1 h at room temperature. Then, the membrane was incubated with primary antibodies [rabbit anti-NDP52 antibody (#ab68588, Abcam, dilution: 1/1000); rabbit anti-optineurin antibody (#ab151240, Abcam, dilution: 1/1000); anti-p62/SQSTM1 (#sc-28359, Santa Cruz Biotechnology, dilution: 1/1000); anti-NBR1 (#20145, Cell Signaling Technology, dilution: 1/1000); anti-TAX1BP1 (#5105, Cell Signaling Technology, dilution: 1/1000); anti-phospho-IκB-α (#2859, Cell Signaling Technology, dilution: 1/1000); anti-β-actin rabbit antibody (#4970, Cell Signaling Technology, dilution: 1/1000)] diluted with the blocking buffer overnight at 4°C. After washes, membranes were incubated with the appropriate HRP-conjugated secondary antibodies (Cell Signaling Technology) in blocking buffer for 1 h at room temperature. After washes, blots were detected using the Clarity Western ECL Substrate (Bio-Rad) and revealed using the ChemiDOcTM XRS System (Bio-Rad).

### Statistical analyses

Results were presented as means ± SEM. Statistical analyses between 2 or several groups were performed using the Student t test (Mann-Whitney if not parametric) or analysis of variance (ANOVA) followed by a post-test Bonferroni correction (Kruskal-Wallis if not parametric), respectively, with GraphPad Prism version 9.4.0 software. A *P* value less than 0.05 was considered statistically significant. *P < 0.05; **P ≤ 0.01; ***P ≤ 0.001; ****P ≤ 0.0001.

## Results

### p62 and NDP52 are autophagy receptors involved in the control of AIEC LF82 intracellular replication

**Figure 1:**
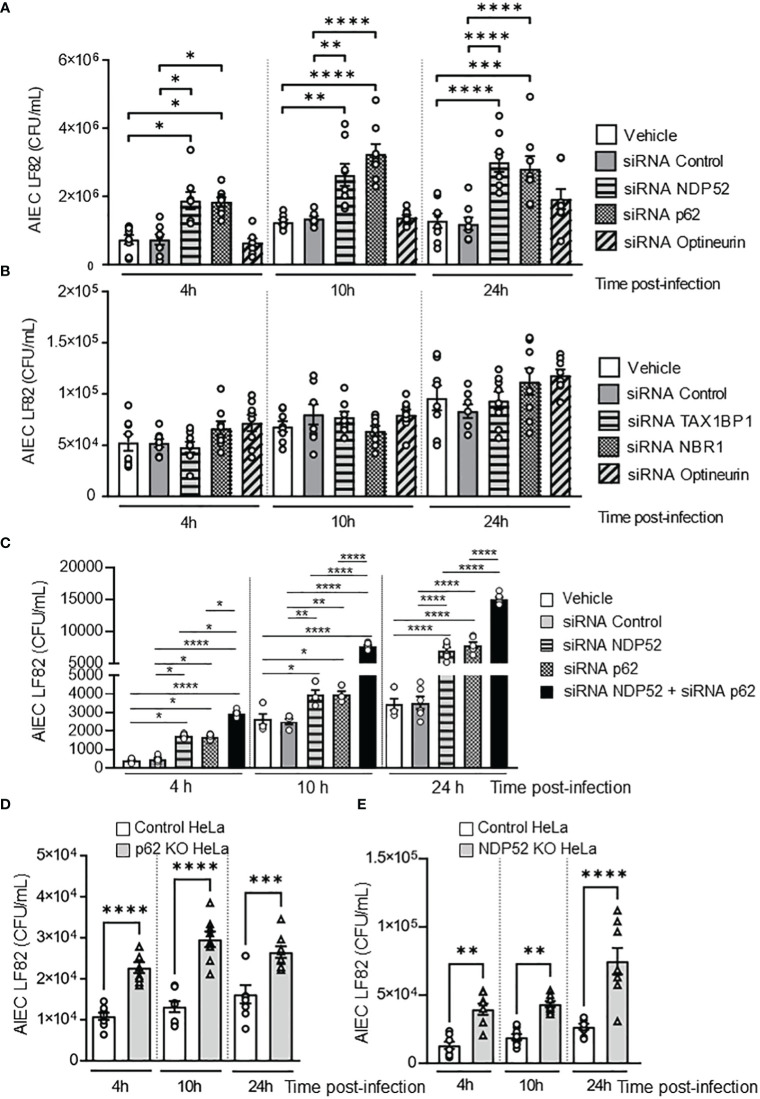
NDP52 and p62 are implicated in the control of AIEC LF82 intracellular number. **(A–C)**. T84 cells were transfected with 50 nM of control siRNA or siRNA against NDP52, p62 or Optineurin **(A)**, or TAX1BP1, NBR1, or Optineurin **(B)**, or with both siRNA against NDP52 and p62 or siRNA against NDP52 or p62 separately **(C)**. 48 h after transfection, the cells were infected with AIEC LF82 strain at a MOI of 10 for 3 h. The cells were then washed and incubated with the infection media containing 100 μg/ml gentamicin for 1, 7 or 21 h, which corresponded to 4, 10 or 24 h post-infection on the graph respectively. The cells were washed, lysed and plated on LB agar plate to determine the colony-forming units of LF82. p62 KO HeLa **(D)** or NDP52 KO HeLa **(E)** cells and their corresponding control cells were infected with the AIEC LF82 strain as in A-C, and the colony-forming units of LF82 were determined. Results are presented as means ± SEM from 3 independent experiments. Different points on the graph presented replicates from 3 independent experiments. Statistical analyses were performed using one-way Anova test followed by a post-test Bonferroni correction. *P < 0.05; **P ≤ 0.01; ***P ≤ 0.001; ****P ≤ 0.0001.

Some autophagy receptors, such as Optineurin, NBR1, TAX1BP1, p62 and NDP52, have been shown to participate in the elimination of invading bacteria (Lamark and Johansen, 2021). To investigate which autophagy receptors are involved in the control of intracellular AIEC LF82 by autophagy, the intestinal epithelial T84 cell line was transfected with siRNAs against Optineurin, NBR1, TAX1BP1, p62 or NDP52. The efficiency of siRNA transfection was analyzed by western blot and was shown in **Supplementary Figure 1**. Among them, a significant increase in the number of intracellular AIEC LF82 was observed in cells transfected with siRNAs against p62 or NDP52, compared to cells transfected with vehicle or a siRNA control at 4, 10 and 24 h post-infection (**Figure 1A**). In the cells transfected with siRNAs against TAX1BP1, Optineurin or NBR1, no significant difference in the AIEC LF82 intracellular number was detected compared to vehicle and siRNA control conditions (**Figures 1A, B**). Together, these results suggest that p62 and NDP52 might be involved in the control of the AIEC LF82 strain by autophagy.

To determine whether p62 and NDP52 could have a synergistic effect to target AIEC, T84 cells were transfected with both siRNAs against p62 and NDP52, and the number of intracellular LF82 in these cells was compared to that in cells transfected with siRNA against p62 or siRNA against NDP52 separately. As shown in **Figure 1C**, a significant increase in the number of intracellular AIEC LF82 in cells transfected with both siRNAs against p62 and NDP52 compared to cells transfected with each siRNA separately was observed at 4, 10 and 24 h post-infection. This result suggested that p62 and NDP52 could have a synergistic effect to target AIEC bacteria.

To confirm the involvement of p62 in autophagy-mediated control of AIEC intracellular number, p62 KO and the control HeLa cells were infected with the AIEC LF82 strain. A significant increase in the number of intracellular AIEC LF82 was observed in p62 KO HeLa cells compared to control cells at 4, 10 et 24 h post-infection (**Figure 1D**). The same experiment was performed using NDP52 KO HeLa cells and their corresponding control cells, and the same results were obtained (**Figure 1E**). Indeed, the intracellular number of AIEC LF82 was significantly higher in NDP52 KO HeLa cells compared to control cells (**Figure 1E**). Thus, these results confirm the involvement of p62 and NDP52 in the control of AIEC LF82 intracellular number.

**Figure 2:**
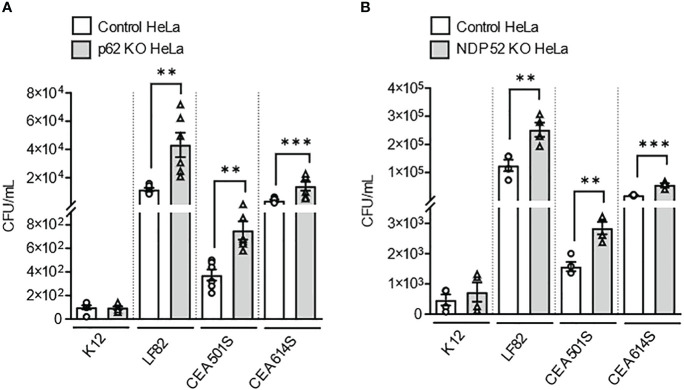
Depletion of NDP52 or p62 leads to enhanced intracellular number of clinical AIEC strains in HeLa cells. P62 KO HeLa **(A)** or NDP52 KO HeLa **(B)** cells and their corresponding control cells were infected with the K12 C600 strain, or different clinical AIEC strains (LF82, CEA501S or CEA614S) at a MOI of 10 for 3 h. The cells were then washed and incubated with the infection media containing 100 μg/ml gentamicin for 1 h. The cells were washed, lysed and plated on LB agar plate to determine the bacterial colony-forming units. Results are presented as means ± SEM from 3 independent experiments. Different points on the graph presented replicates from 3 independent experiments. Statistical analyses were performed using one-way Anova test followed by a post-test Bonferroni correction. **P ≤ 0.01; ***P ≤ 0.001.

Next, to further investigate the role of these two receptors in the recognition of other clinical AIEC strains, p62 KO HeLa cells, NDP52 KO HeLa cells and their respective control cells were infected with two other clinical AIEC strains (CEA501S and CEA614S) isolated from the ileal mucosa of CD patients [CEALIVE cohort (Buisson et al., 2021)]. The non-pathogenic and non-invasive *E. coli* K12 C600 strain and the AIEC LF82 strain were also used. In p62 KO HeLa cells, the intracellular number of the AIEC CEA501S and CEA614S strains was significantly higher compared to that in control cells at 4h post-infection (**Figure 2A**). This was also observed for the AIEC LF82 strain but not the K12 C600 strain (**Figure 2A**). Similarly, the intracellular number of the CEA501S and CEA614S strains was significantly higher in NDP52 KO HeLa cells compared to control cells at 4h post-infection (**Figure 2B**). This increase was also observed for the AIEC LF82 strain, but not the K12 C600 strain (**Figure 2B**). Taken together, these results suggest the role of p62 and NDP52 in controlling the intracellular number of clinical AIEC strains.

### Direct colocalization between p62 or NDP52 with the AIEC LF82 strain and the autophagic protein LC3

**Figure 3:**
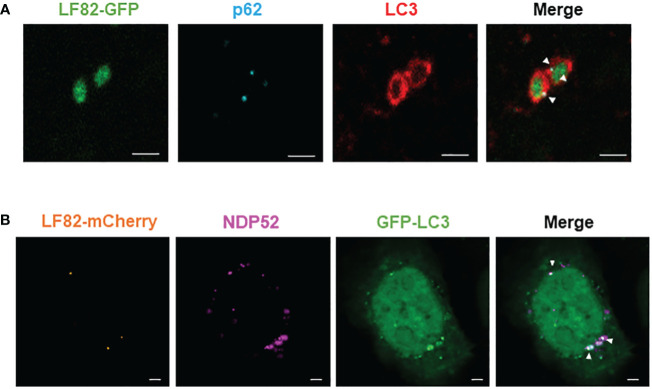
NDP52 and p62 directly colocalize with AIEC LF82 bacteria and the autophagic LC3 protein in HeLa cells. **(A)** HeLa cells were infected with AIEC LF82-GFP at a MOI of 100 for 3 h, washed and incubated with 100 μg/ml gentamicin for 3 h. Immunofluorescent labeling to detect p62 (blue) and LC3 (red) was performed. **(B)** HeLa-GFP-LC3 cells were infected with AIEC LF82-mCherry at a MOI of 100 for 3 h, washed and incubated with 100 μg/ml gentamicin for 3 (h). Immunofluorescent labeling to detect NDP52 (magenta) was performed. Observation was performed on a Zeiss LSM 800 Airyscan confocal microscope. Each experiment was repeated 3 times, and 2 replicates (2 coverslips) were prepared for each experiment. For each coverslip, 20 images were taken. Bars: 2 μm.

It has been shown that autophagy receptors can bind the autophagic protein LC3 via their LC3-binding domain and can also bind ubiquitin on the bacterial surface via their ubiquitin-binding domain (Lamark and Johansen, 2021). To demonstrate that p62 and NDP52 act as autophagy receptors for AIEC, we performed immunofluorescent staining to observe a colocalization between NDP52 or p62 with AIEC LF82 bacteria and the autophagic protein LC3. **Figure 3A** shows a direct colocalization between LF82-GFP bacteria, p62 (blue) and LC3 (red). We also observed a colocalization between LF82-mCherry, NDP52 (magenta) and GFP-LC3 (**Figure 3B**). Together, these results demonstrate that p62 and NDP52 are autophagy receptors responsible for the recognition and targeting AIEC LF82 bacteria to LC3-positive autophagosome.

Data for bacterial invasion assay performed with the same MOI and time post-infection used for immunofluorescent staining was shown in **Supplementary Figure 2**. This result showed that depletion in p62 or NDP52 led to increase in intracellular number of the AIEC LF82 strain.

### NDP52 and p62 are required to control AIEC LF82-induced inflammation

**Figure 4:**
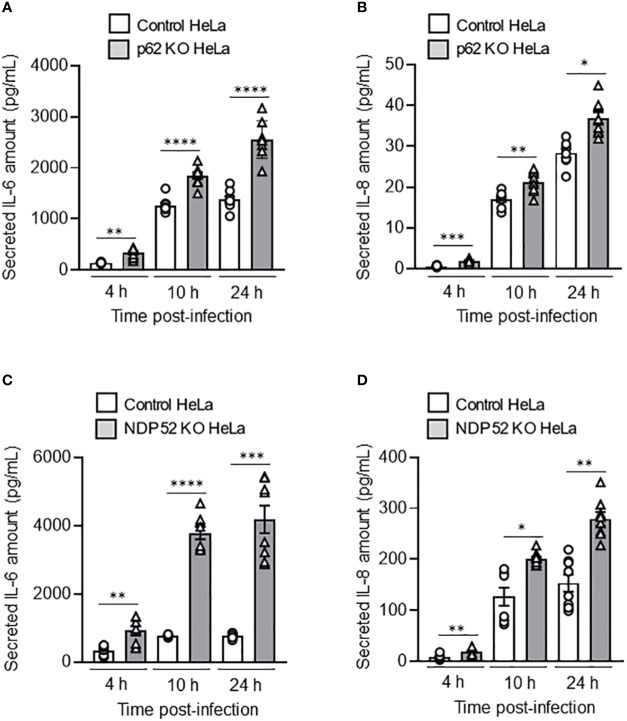
Depletion of NDP52 or p62 leads to increased AIEC LF82-induced pro-inflammatory cytokine production in HeLa cells. p62 KO **(A, B)** or NDP52 KO **(C, D)** HeLa cells and their corresponding control cells were infected with AIEC LF82 strain at a MOI of 10 for 3 h. The cells were then washed and incubated with the infection media containing 100 μg/ml gentamicin for 1, 7 or 21 h, which corresponded to 4, 10 or 24 h post-infection on the graphs respectively. Cell culture supernatants were collected at 4, 10 and 24 h post-infection, and the amount of secreted IL-6 **(A, C)** and IL-8 **(B, D)** were analyzed by ELISA. Results are presented as means ± SEM from 3 independent experiments. Different points on the graph presented replicates from 3 independent experiments. Statistical analyses were performed using one-way Anova test followed by a post-test Bonferroni correction. *P < 0.05; **P ≤ 0.01; ***P ≤ 0.001; ****P ≤ 0.0001.

It has been shown that AIEC LF82 bacteria are able to induce the production of pro-inflammatory cytokines by host cells (Nguyen et al., 2014; Bretin et al., 2016). Thus, we sought to analyze the role of NDP52 and p62 in the control of AIEC-induced pro-inflammatory cytokine production. In p62 KO HeLa cells, a significant increase in the amount of secreted IL-6 and IL-8 was detected at 4h, 10h and 24h after LF82 infection at a MOI of 10 compared to control cells (**Figures 4A, B**). Similarly, the amount of secreted IL-6 and IL-8 was increased in NDP52 KO HeLa cells compared to control cells after 4h, 10h and 24h of LF82 infection at a MOI of 10 (**Figures 4C, D**). Similar results were obtained under LF82 infection at a MOI of 100 (**Supplementary Figure 3**). Together, these results showed that p62 and NDP52 are also required to limit AIEC LF82-induced pro-inflammatory cytokine production.

### The CD-associated *NDP52^Val248Ala^* variant does not impact AIEC LF82 intracellular replication in HeLa cells

**Figure 5:**
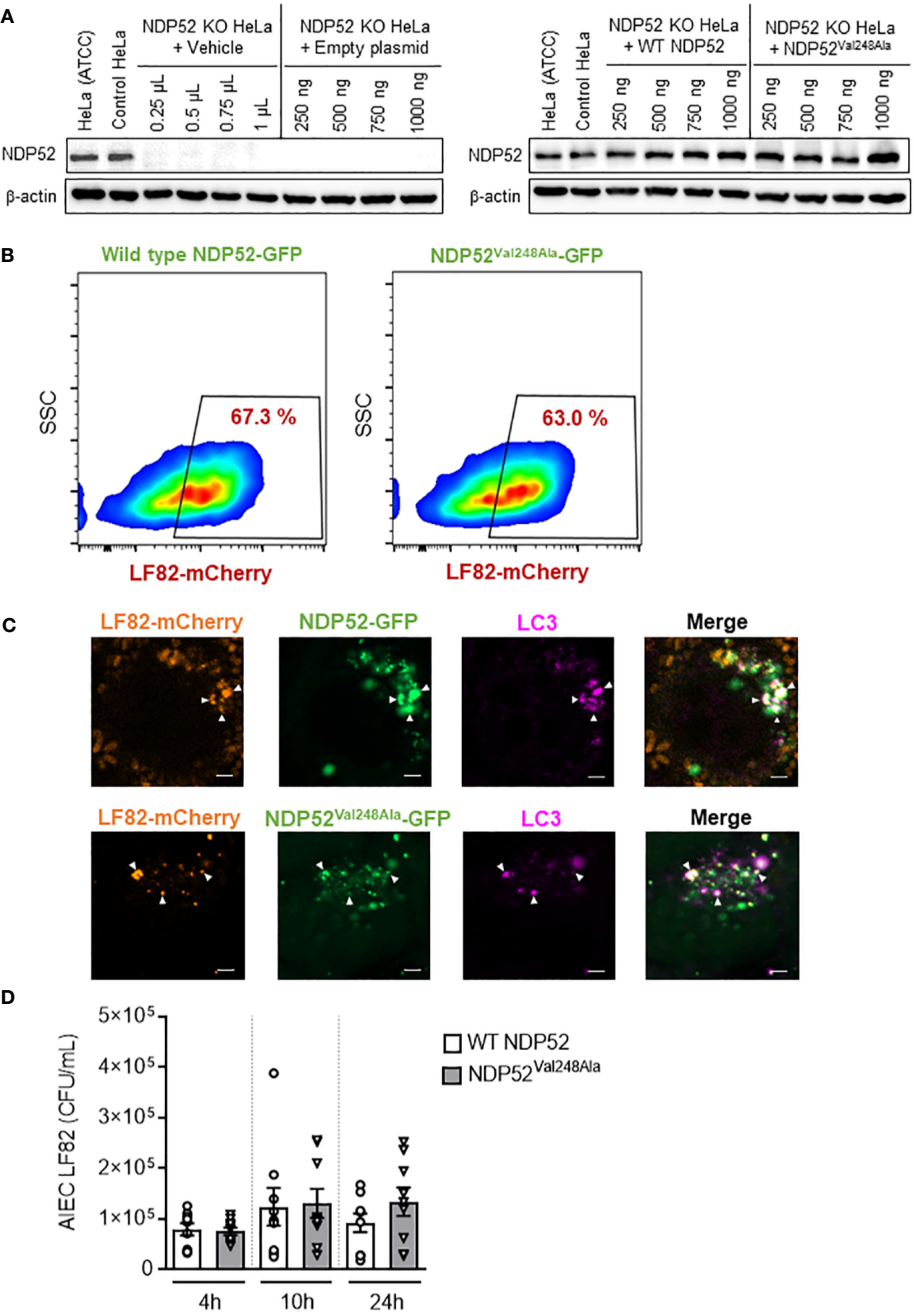
The CD-associated *NDP52^Val248Ala^* variant does not impact AIEC LF82 intracellular number in HeLa cells. **(A)** Western blot analysis of NDP52 expression in NDP52 KO HeLa cells transfected with different quantities (250, 500, 750 or 1000 ng/well) of a construct that expresses wild-type NDP52 or the mutated NDP52^Val248Ala^ or the empty plasmid or with different volumes of vehicle (corresponding to the volumes of plasmid added). HeLa cells from ATCC and the corresponding control cells of NDP52 KO cells were used in parallel. **(B, C)** NDP52 KO HeLa cells expressing NDP52-GFP or NDP52^Val248Ala^-GFP were infected with AIEC LF82-mCherry at a MOI of 100 for 3 h. The cells were then washed and incubated with the infection media containing 100 μg/ml gentamicin for 3 h. **(B)** The percentage of mCherry-positive cells (LF82-infected cells) among GFP-positive cells (transfected cells) was determined by flow cytometry. **(C)** Immunofluorescent labeling to detect LC3 (magenta) was performed. Observation was performed on a Zeiss LSM 800 Airyscan confocal microscope. Each experiment was repeated 3 times, and 2 replicates (2 coverslips) were prepared for each experiment. For each coverslip, 20 images were taken. Bars: 2 μm. **(D)** NDP52 KO HeLa cells transfected with the construct expressing wild-type NDP52 or NDP52^Val248Ala^ protein were infected with AIEC LF82 at a MOI of 100 for 3 h. The cells were then washed and incubated with the infection media containing 100 μg/ml gentamicin for 1, 7 or 21 h, which corresponded to 4, 10 or 24 h post-infection on the graphs, respectively. The cells were washed, lysed and plated on LB agar plates to determine the bacterial colony-forming units (CFUs). Results are presented as means ± SEM from 3 independent experiments.

As the *NDP52^Val248Ala^* polymorphism has been associated with an increased susceptibility to develop CD (Ellinghaus et al., 2013), we investigated its potential impact on host response to AIEC infection. For this, NDP52 KO HeLa cells were transfected with a construct that expresses the wild-type NDP52 or the mutated NDP52^Val248Ala^ protein. Western blot analysis showed that the level of NDP52 protein in these cells were similar (**Figure 5A**). We also examined whether expression of the *NDP52^Val248Ala^* risk variant modifies expression of p62. As shown in **Supplementary Figure S4**, p62 protein level was similar between HeLa cells expressing the wild-type NDP52 and those expressing the mutated NDP52^Val248Ala^ protein.

A flow cytometry experiment was performed to investigate the impact of the mutated NDP52^Val248Ala^ protein on the control of AIEC LF82 infection by host cells. For this, NDP52 KO HeLa cells were transfected with the GFP plasmid expressing wild-type NDP52 or the mutated NDP52^Val248Ala^ protein, and infected with the AIEC LF82-mCherry bacteria. Then, the percentage of mCherry-positive cells (LF82-infected cells) among GFP-positive cells (transfected cells) was determined by flow cytometry. This allowed to select only the transfected cells, and to count the transfected cells that are infected with LF82-mCherry. Our results showed that 67.3% of the cells expressing wild-type NDP52-GFP were infected, and 63% of the cells expressing NDP52^Val248Ala^-GFP were infected (**Figure 5B**). The gating strategy was represented in **Supplementary Figure 5**, and all control conditions (transfected with empty plasmid, uninfected) were represented in **Supplementary Figure 6**.

This result suggested that expression of the mutated NDP52^Val248Ala^ protein did not seem to impact the percentage of LF82-infected cells. Furthermore, fluorescent microscopy analysis showed that the mutated NDP52^Val248Ala^-GFP protein was able to bind to the LF82-mCherry bacteria and the autophagic protein LC3 (magenta) as the wild-type NDP52 did (**Figure 5C**). Finally, we investigated the impact of the *NDP52^Val248Ala^* variant on AIEC LF82 intracellular replication by gentamicin protection assay. No significant difference in the AIEC LF82 intracellular number at 4, 10 and 24 h post-infection was observed between HeLa cells expressing the wild-type NDP52 or the mutated NDP52^Val248Ala^ protein (**Figure 5D**). Together, these results suggested that in HeLa cells, the CD-associated *NDP52^Val248Ala^* polymorphism has no impact on AIEC recognition and targeting to autophagy, thus does not influence the control of AIEC intracellular replication.

### The CD-associated *NDP52^Val248Ala^* variant does not impact AIEC-induced NF-κB activation and pro-inflammatory cytokine production in HeLa cells

**Figure 6:**
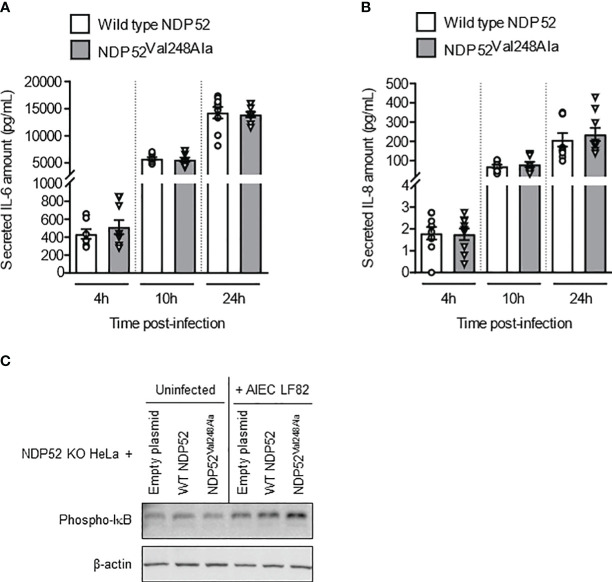
The CD-associated *NDP52^Val248Ala^* variant does not impact AIEC LF82-induced NF-κB activation and pro-inflammatory cytokine production in HeLa cells. NDP52 KO HeLa cells were transfected with a construct that expresses wild-type NDP52 or the mutated NDP52^Val248Ala^ **(A–C)**, and were infected with AIEC LF82 at a MOI of 10 for 3 h. The cells were then washed and incubated with the infection media containing 100 μg/ml gentamicin for 1, 7 or 21 h (which corresponded to 4, 10 or 24 h post-infection on the graphs respectively) **(A, B)** or 3 h **(C)**. **(A, B)** Cell culture supernatants were collected at 4, 10 and 24 h post-infection, and the amounts of secreted IL-6 and IL-8 were analyzed by ELISA. Results are presented as means ± SEM from 3 independent experiments. Different points on the graph represented replicates from 3 independent experiments. **(C)** Western blot analysis for phospho-IκB levels. The immunoblot is representative of three independent experiments..

To further examine the impact of the CD-associated *NDP52^Val248Ala^* polymorphism on the control of AIEC LF82-induced inflammation, NDP52 KO HeLa cells were transfected with the construct expressing the wild-type NDP52 or the mutated NDP52^Val248Ala^ protein, and infected with the AIEC LF82 strain with a MOI of 10 (**Figure 6**) or 100 or 200 (**Supplementary Figure 7**). No significant difference was observed in the amount of IL-6 and IL-8 cytokines secreted by HeLa cells expressing the wild-type NDP52 and those expressing the NDP52^Val248Ala^ protein (**Figures 6A, B**). Furthermore, Western blot analysis showed a similar level of phospho-IκB in cells expressing the mutated NDP52^Val248Ala^ protein compared to those expressing the wild-type NDP52 under both uninfected and LF82-infected conditions (**Figure 6C**). These results suggested that the CD-associated *NDP52^Val248Ala^* polymorphism has no impact on AIEC-induced inflammation in HeLa cells.

## Discussion and conclusion

Xenophagy is an important mechanism of selective autophagy allowing the degradation of intracellular pathogens. This specific elimination is ensured by autophagy receptors which act as a bridge between the cargo to be degraded and the LC3 molecules on autophagosome (Lamark and Johansen, 2021). Our group has shown that xenophagy is a key mechanism of host cells to eliminate intracellular AIEC bacteria, which have emerged as an important player in the etiopathogenesis of CD (Larabi et al., 2020a). However, the receptor(s) that recognize AIEC and targeting the bacteria to autophagy have not been elucidated.

In this study, we demonstrated that NDP52 and p62 act as autophagy receptors for AIEC in epithelial cells, therefore limiting AIEC intracellular number. Indeed, we showed that NDP52 and p62 receptors directly colocalized with intracellular AIEC LF82 bacteria and with LC3, a key protein for autophagosome formation (Klionsky et al., 2021). Furthermore, the depletion of NDP52 or p62 in epithelial cells led to an increase in the intracellular number of AIEC LF82 as well as other clinical AIEC strains. In line with our results, it has been shown that these two autophagy receptors are involved in the recognition of other intracellular bacteria, such as *Listeria monocytogenes* (Mostowy et al., 2011), *Shigella flexneri* (Mostowy et al., 2011) and *Salmonella enterica* serovar Typhimurium (Thurston et al., 2009; Zheng et al., 2009). It has been highlighted that the same autophagy receptor can target different bacteria into different autophagosomes (Sharma et al., 2018). For example, Mostowy and co-workers showed that NDP52 and p62 target *Shigella flexneri* to autophagosomes in an actin-septin dependent manner, whereas they target *Listeria monocytogenes* to autophagosome via an autophagy pathway independently of septin or actin (Mostowy et al., 2011). Additionally, it has been shown that NDP52 and p62 can bind to distinct micro-domains of bacteria (Cemma et al., 2011). These studies reinforce the fact that different pathogens can induce different pathways of selective autophagy (Sharma et al., 2018), and thus the elucidation of autophagy receptors for each pathogen is of importance and needs to be further investigated.

As we have previously shown that AIEC can induce the production of pro-inflammatory cytokines by host epithelial cells (Nguyen et al., 2014; Bretin et al., 2016), we further investigated the role of NDP52 and p62 in controlling AIEC-induced inflammation. Our results highlighted that the depletion of NDP52 or p62 resulted in enhanced IL-6 and IL-8 secretion by epithelial cells upon AIEC infection. This indicates that NDP52 and p62 are required to control AIEC-induced inflammation.

Interestingly, a whole exome sequencing study identified an association between increased susceptibility to develop CD and a common missense variant, Val248Ala (SNP rs2303015), which leads to the replacement of valine by alanine at the position 248 of NDP52 protein (Ellinghaus et al., 2013). However, the exact implication of the *NDP52^Val248Ala^* risk variant in CD etiopathogenesis remained a mystery. We hypothesized that this risk variant could lead to a defect in the control of AIEC intracellular number, leading to AIEC abnormal replication and enhanced AIEC-induced inflammation, and this could be a potential mechanism by which the *NDP52^Val248Ala^* variant may contribute to CD etiopathogenesis. Our results showed that the *NDP52^Val248Ala^* risk variant does not appear to impact the control of AIEC LF82 by autophagy as the mutated NDP52^Val248Ala^ protein still colocalized with LF82 bacteria and LC3 as did the wild-type NDP52 protein. Furthermore, in HeLa cells, under our experimental conditions, expression of the mutated NDP52^Val248Ala^ protein did not influence neither the replication of LF82 bacteria nor the LF82-induced NF-κB activation and pro-inflammatory cytokine production. These results suggest that the *NDP52^Val248Ala^* variant may be implicated in CD etiopathogenesis via another mechanism, such as regulation of inflammation, rather than via impacting the autophagy-mediated control of AIEC colonization.

Indeed, the protein NDP52 has been shown to be able to reduce inflammation via down-regulating the NF-κB signaling (Inomata et al., 2012; Ellinghaus et al., 2013). Ellinghaus and colleagues reported that while the wild-type NDP52 was able to decrease NF-κB activation in response to the TLR agonist poly(I:C) in HeLa cells, the CD-associated NDP52^Val248Ala^ protein failed to do so (Ellinghaus et al., 2013). The authors thus proposed that under physiological state, wild-type NDP52 could selectively degrade TLR adaptors or other PAMP (Pathogen-associated molecular pattern) receptors, inhibiting activation of the pro-inflammatory NF-κB signaling pathway. However, in pathological state, such as in CD, the *NDP52^Val248Ala^* risk variant may fail to recognize polyubiquitinated TLR adaptors, inducing adaptor stabilization and consequently high NF-κB activity, thereby causing aggravated inflammation as observed in CD patients (Ellinghaus et al., 2013). The discrepancy between this work and our results could be explained by the fact that, contrary to Ellinghaus and co-authors who used poly(I:C) to induce activation of the NF-κB pathway, we used an AIEC infection model. In our AIEC-infected HeLa cell model, the impact of wild-type NDP52 and mutated NDP52^Val248Ala^ protein on inflammation was similar. But we could imagine that in specific pathological condition, the charge of AIEC could be different compared to our experimental condition, and under these conditions, NF-κB activation might be markedly increased, and cannot be down-regulated by the mutated NDP52^Val248Ala^ protein. The implication of the *NDP52^Val248Ala^* risk variant in CD etiopathogenesis thus needs to be further investigated using different models of experimentation.

In conclusion, our results demonstrated that NDP52 and p62 are autophagy receptors responsible for the targeting of AIEC to autophagy pathway, thus controlling AIEC intracellular replication and AIEC-induced inflammation.

## Data availability statement

The original contributions presented in the study are included in the article/**Supplementary Material**. Further inquiries can be directed to the corresponding authors.

## Author contributions

AD: Data curation, Formal analysis, Methodology, Writing – original draft. GD: Formal analysis, Methodology, Writing – review & editing. AL: Formal analysis, Methodology, Writing – review & editing. MH: Formal analysis, Writing – review & editing. EB: Formal analysis, Writing – review & editing. NB: Writing – review & editing, Methodology, Conceptualization, Funding acquisition, Supervision. HN: Conceptualization, Funding acquisition, Methodology, Supervision, Writing – review & editing, Data curation, Formal analysis, Investigation, Resources, Software, Validation, Visualization, Writing – original draft.
