# Supplementary material for: Identification of autophagy receptors for the Crohn’s disease-associated adherent-invasive Escherichia coli
Source: Front Cell Infect Microbiol. 2024 Mar 28;14:1268243. doi: 10.3389/fcimb.2024.1268243 (PMC11007067; doi:10.3389/fcimb.2024.1268243)
Supplement: Supplementary file 1 [file DataSheet_1.pdf]

Identification of autophagy receptors for the Crohn's disease-associated adherent-invasive *Escherichia coli*

Alison Da Silva[1], Guillaume Dalmasso[1], Anaïs Larabi[1], My Hanh Thi Hoang[1,2], Elisabeth Billard[1], Nicolas Barnich[1,#,*], Hang Thi Thu Nguyen[1,#,*]

[1]M2iSH (Microbes, intestine, inflammation and Susceptibility of the Host), UMR 1071 Inserm, Université Clermont Auvergne, INRAe USC 1382, CNRH, 63001 Clermont-Ferrand, France.

[2]Department of Cell Biology, Faculty of Biology, University of Science, Vietnam National University, Hanoi, Vietnam.

[#]These authors contributed equally to this work

*Correspondences: nicolas.barnich@uca.fr or hang.nguyen@uca.fr.

Tel.: (+33)473178345; Fax: (+33)473178371.

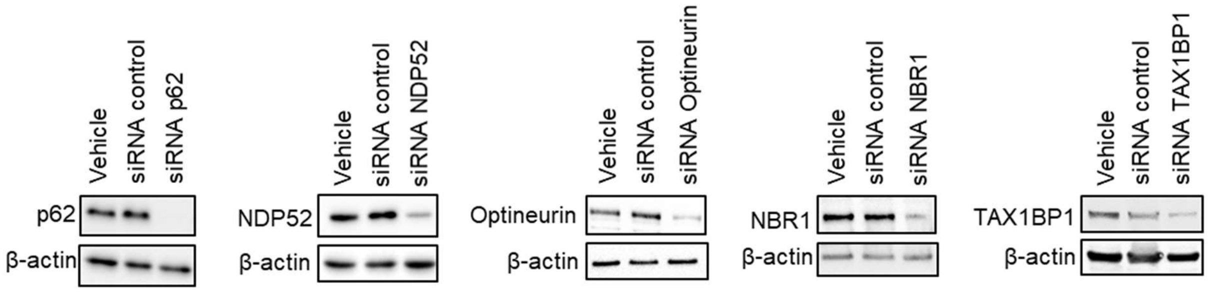

Figure S1: Efficiency of transfection of T84 cells with siRNAs.

T84 cells were transfected with vehicle or 50 nM of control siRNA or siRNA against NDP52, p62, Optineurin, NBR1 or TAX1BP1. 48 h after transfection, the cells were lysed, and western blot analysis was performed to verify the efficiency of siRNA transfection.

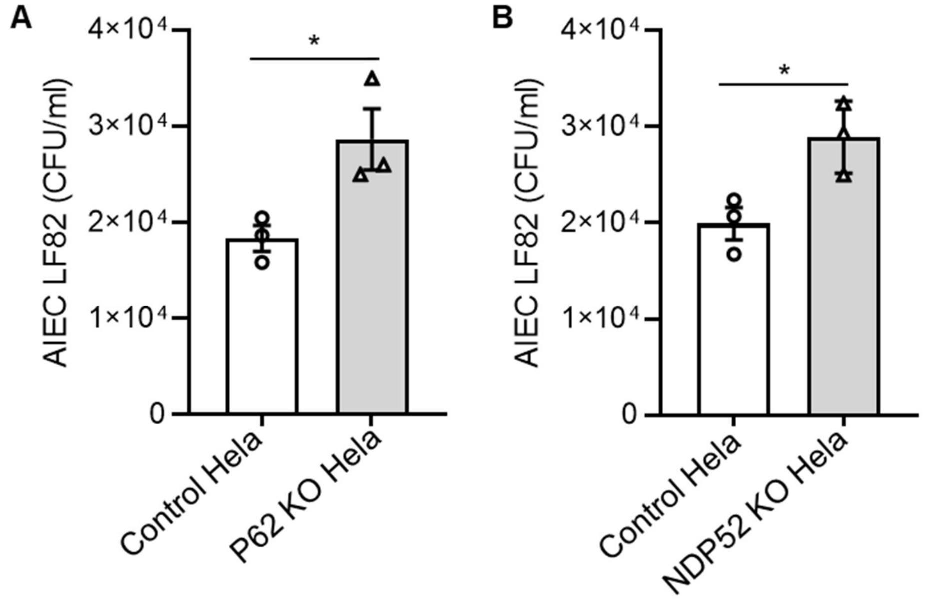

Figure S2: Depletion of NDP52 or p62 leads to increased AIEC LF82 intracellular number in HeLa cells at 6 h post-infection.

p62 KO or NDP52 KO HeLa cells and their corresponding control cells were infected with the AIEC LF82 strain at a MOI of 100 for 3 h. The cells were then wash and incubated with the infection media containing 100 µg/ml gentamicin for 3 h. The cells were washed, lysed and plated on LB agar plate to determine the bacterial colony-forming units. Results are presented as means ± SEM from 3 independent experiments. Statistical analyses were performed using unpaired t-test. $*P < 0.05$.

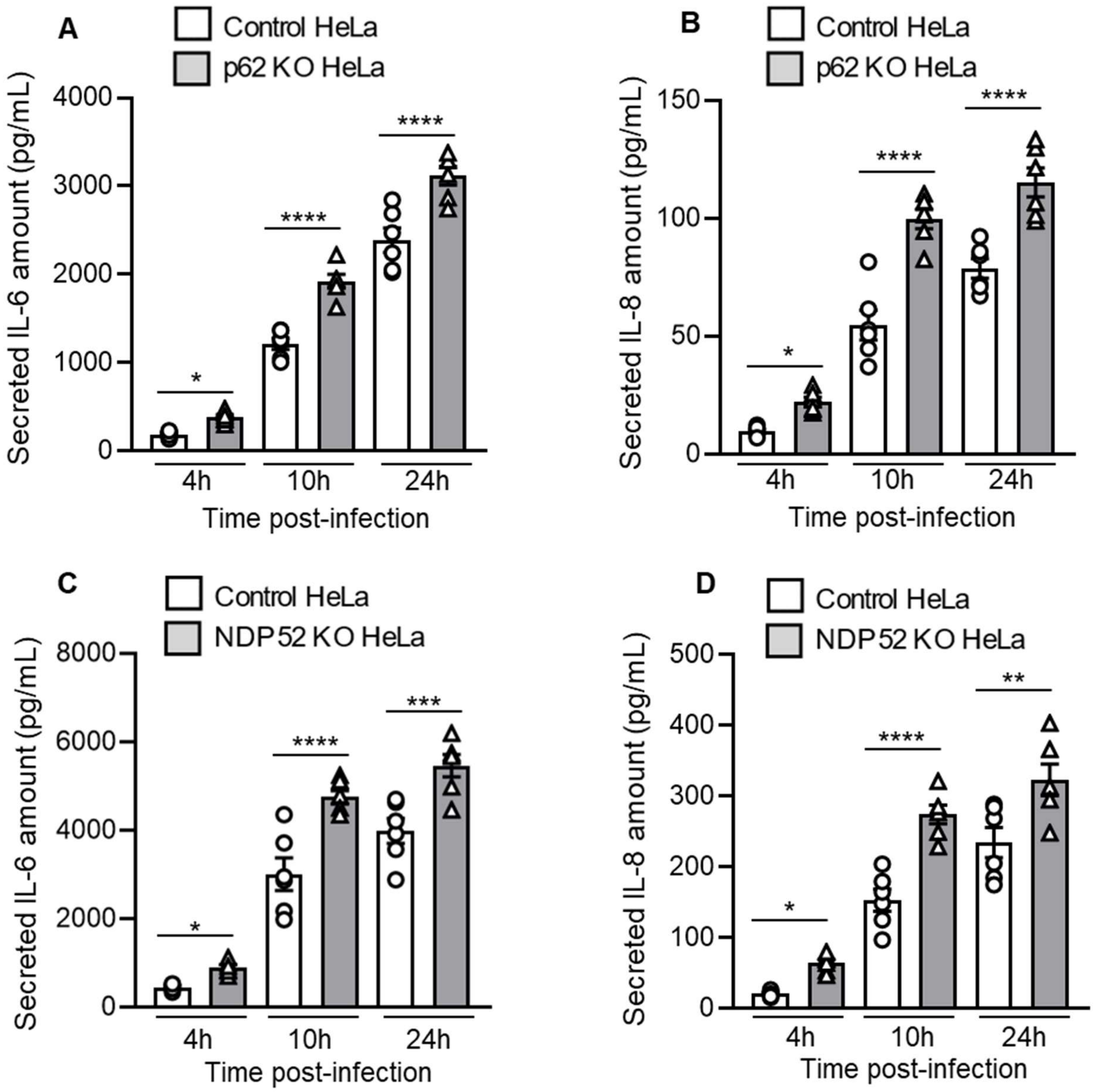

Figure S3: Depletion of NDP52 or p62 leads to increased AIEC LF82-induced pro-inflammatory cytokine production in HeLa cells.

p62 KO (**A, B**) or NDP52 KO (**C, D**) HeLa cells and their corresponding control cells were infected with AIEC LF82 strain at a MOI of 100 for 3 h. The cells were then wash and incubated with the infection media containing 100 μg/ml gentamicin for 1, 7 or 21 h, which corresponded to 4, 10 or 24 h post-infection on the graphs respectively. Cell culture supernatants were collected at 4, 10 and 24 h post-infection, and the amount of secreted IL-6 (**A, C**) and IL-8 (**B, D**) were analyzed by ELISA. Results are presented as means ± SEM from 3 independent experiments. Statistical analyses were performed using one-way Anova test followed by a post-test Bonferroni correction. *$P < 0.05$; **$P \leq 0.01$; ***$P \leq 0.001$; ****$P \leq 0.0001$.

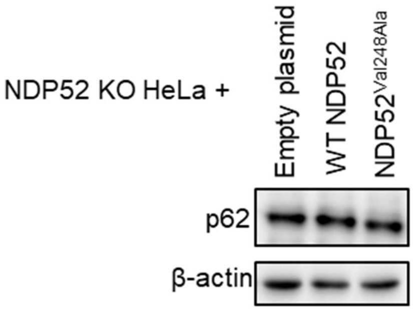

Figure S4: Expression of the CD-associated *NDP52^{Val248Ala}* variant does not impact p62 protein level in HeLa cells.

NDP52 KO HeLa cells were transfected with 500 ng/well of a construct that expresses wild-type NDP52 or the mutated NDP52^{Val248Ala} or the empty plasmid, and p62 expression was analysed by Western blot 2 days post-transfection.

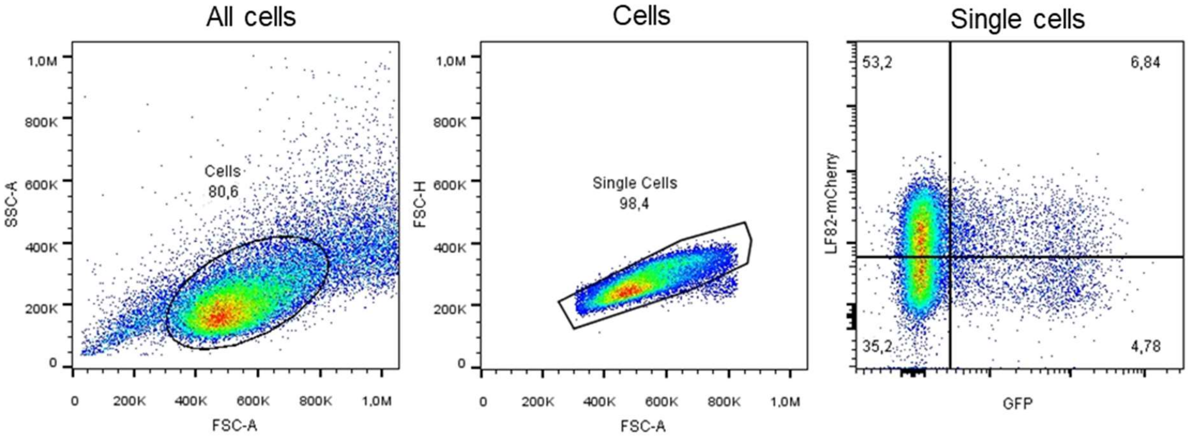

Figure S5: Gating strategy for flow cytometry experiments. Cells were selected on FSC-A vs SSC-A properties and doublets were excluded by plotting FSC-A vs FSC-H before analysis of GFP (plasmid-encoded) *versus* LF82-mCherry fluorescence.

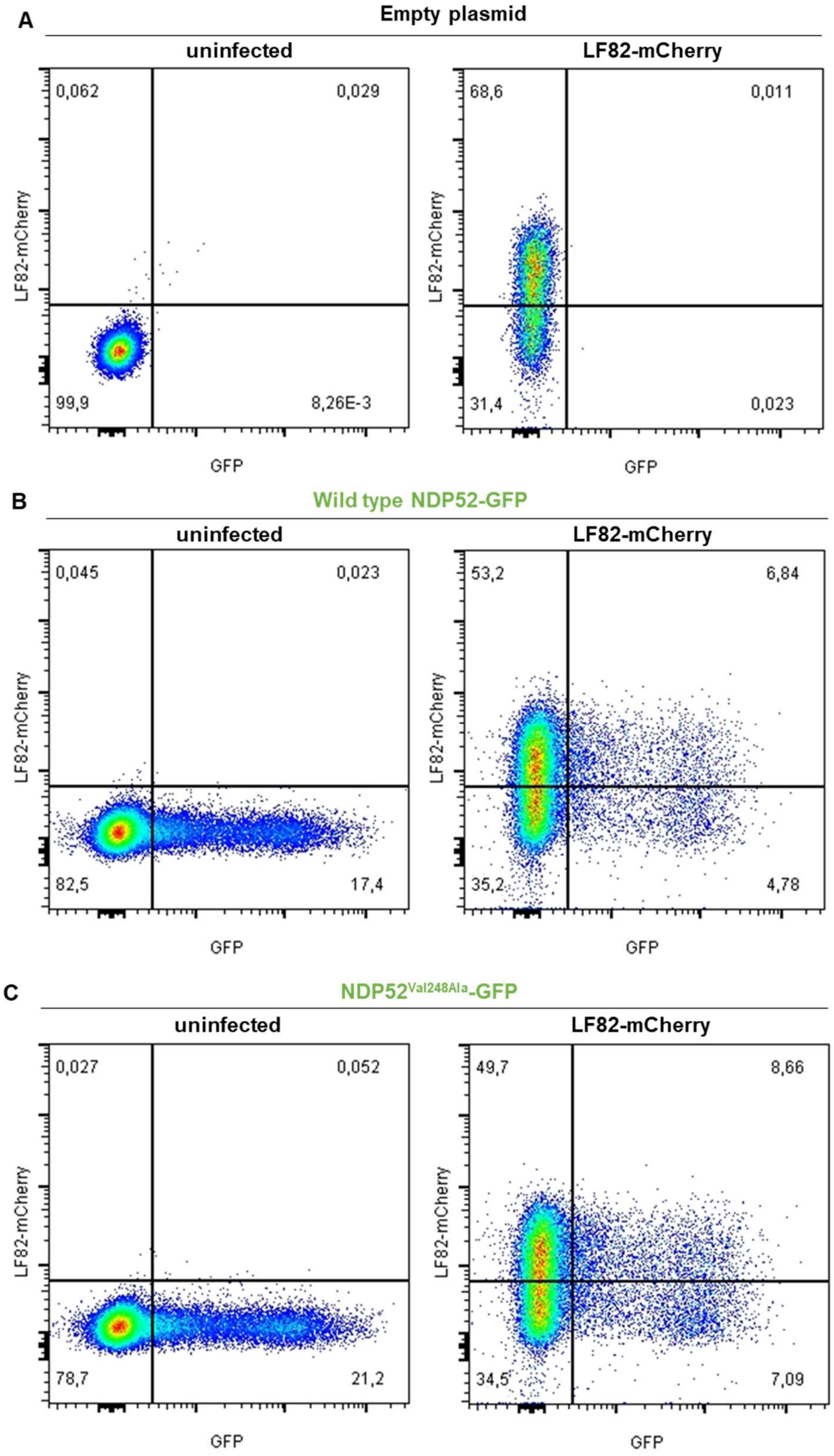

Figure S6. Analysis of mCherry-positive (LF82-infected) and GFP-positive (transfected) cells by flow cytometry.

NDP52 KO HeLa cells expressing NDP52-GFP or NDP52^{Val248Ala}-GFP were infected with LF82-mCherry at a MOI of 100 for 3 h. The cells were then washed and incubated with 100 µg/ml gentamicin for 3 h. GFP and mCherry fluorescence were assessed by flow cytometry.

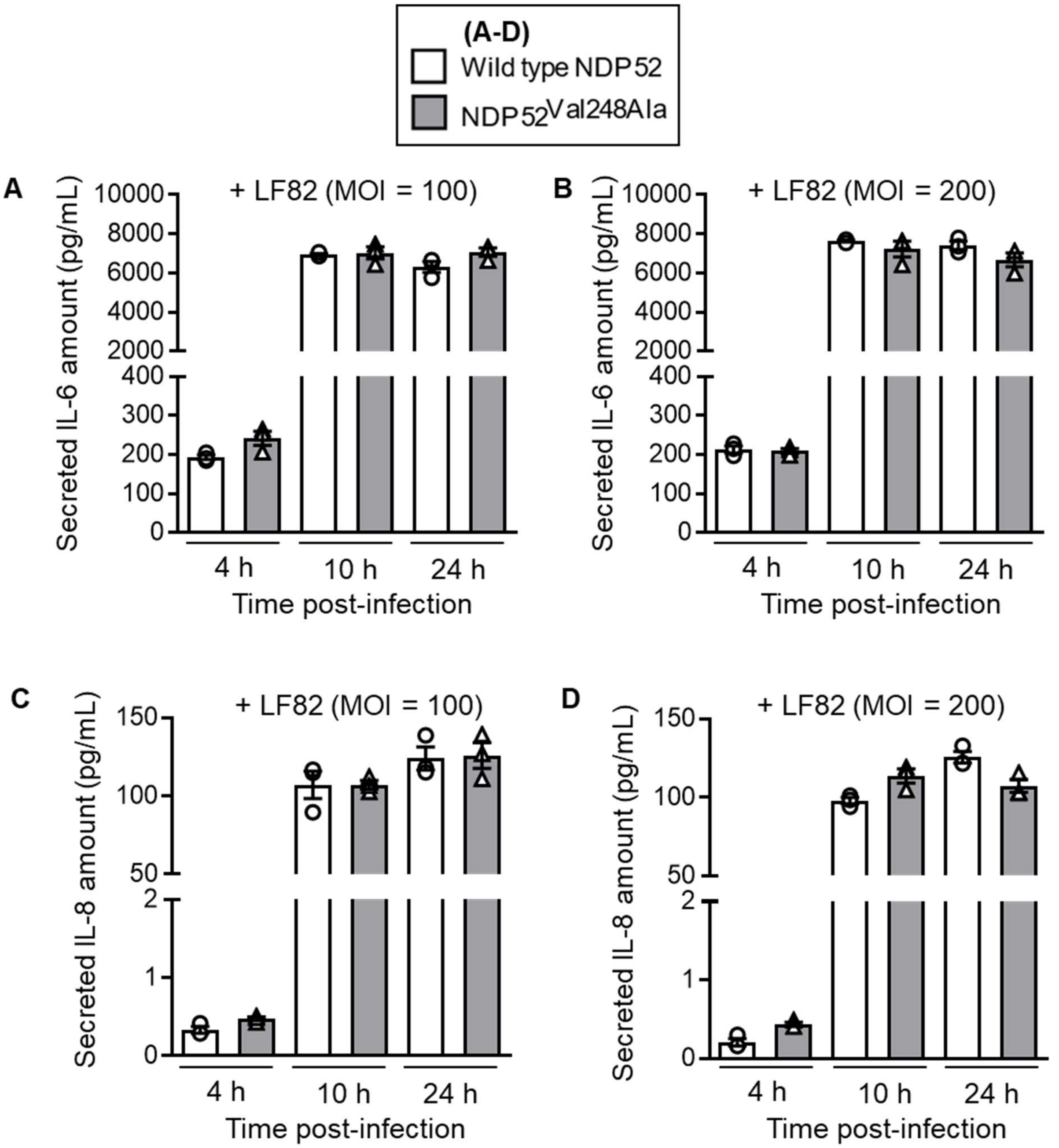

Figure S7: The CD-associated *NDP52^{Val248Ala}* variant does not impact AIEC LF82-induced pro-inflammatory cytokine production in HeLa cells.

NDP52 KO HeLa cells were transfected with a construct that expresses wild-type NDP52 or the mutated NDP52^{Val248Ala}, and were infected with AIEC LF82 at a MOI of 100 (**A, C**) or 200 (**B, D**) for 3 h. The cells were then washed and incubated with the infection media containing 100 µg/ml gentamicin for 1, 7 or 21 h (which corresponded to 4, 10 or 24 h post-infection on the graphs respectively). Cell culture supernatants were collected at the indicated time, and the amounts of secreted IL-6 (**A, B**) and IL-8 (**C, D**) were analyzed by ELISA. Results are presented as means ± SEM from 3 independent experiments.
